# Functionalized ZnO Nanoparticles with Gallic Acid for Antioxidant and Antibacterial Activity against Methicillin-Resistant *S. aureus*

**DOI:** 10.3390/nano7110365

**Published:** 2017-11-02

**Authors:** Joo Min Lee, Kyong-Hoon Choi, Jeeeun Min, Ho-Joong Kim, Jun-Pil Jee, Bong Joo Park

**Affiliations:** 1 Department of Food and Nutrition, Chosun University, Gwangju 61452, Korea; joominlee@chosun.ac.kr; 2 Institute of Biomaterials, Kwangwoon University, 20 Kwangwoongil, Nowon-gu, Seoul 01897, Korea; solidchem@hanmail.net; 3 Halla Energy and Environment, Seoul 05769, Korea; iseeiget@naver.com; 4 Department of Chemistry, Chosun University, Gwangju 61452, Korea; 5 College of Pharmacy, Chosun University, Gwangju 61452, Korea; 6 Department of Electrical & Biological Physics, Kwangwoon University, 20 Kwangwoongil, Nowon-gu, Seoul 01897, Korea

**Keywords:** ZnO@GA, antioxidative activity, antibacterial activity, gallic acid, antibiotic resistance, methicillin-resistant *Staphylococcus aureus*

## Abstract

In this study, we report a new multifunctional nanoparticle with antioxidative and antibacterial activities in vitro. ZnO@GA nanoparticles were fabricated by coordinated covalent bonding of the antioxidant gallic acid (GA) on the surface of ZnO nanoparticles. This addition imparts both antioxidant activity and high affinity for the bacterial cell membrane. Antioxidative activities at various concentrations were evaluated using a 2,2′-azino-bis(ethylbenzthiazoline-6-sulfonic acid) (ABTS) radical scavenging method. Antibacterial activities were evaluated against Gram-positive bacteria (*Staphylococcus aureus: S. aureus*), including several strains of methicillin-resistant *S. aureus* (MRSA), and Gram-negative bacteria (*Escherichia coli*). The functionalized ZnO@GA nanoparticles showed good antioxidative activity (69.71%), and the bactericidal activity of these nanoparticles was also increased compared to that of non-functionalized ZnO nanoparticles, with particularly effective inhibition and high selectivity for MRSA strains. The results indicate that multifunctional ZnO nanoparticles conjugated to GA molecules via a simple surface modification process displaying both antioxidant and antibacterial activity, suggesting a possibility to use it as an antibacterial agent for removing MRSA.

## 1. Introduction

Infectious diseases caused by bacteria are a significant burden on public health and threaten the economic stability of societies worldwide [1]. In particular, the widespread incorrect use of conventional antibiotics has led to the adaptation of microorganisms to these therapies, and the appearance of antibiotic-resistant bacteria is a serious problem [2,3]. Currently, MRSA is the most commonly found antibiotic-resistant bacteria in many parts of the world [4]. Over the last decade, MRSA strains have become one of the main causes of mortality among hospital-acquired infectious diseases [5]. However, the development of novel antibiotics to solve this problem had limited progress. Therefore, the development of alternative methods to treat infections caused by antibiotic-resistant bacteria is an urgent challenge in medical biotechnology.

Material science researchers have focused on designing multifunctional nanoparticles, which combine various functionalities such as fluorescence, magnetism, and photoactivation to generate reactive oxygen species (ROS), and biotargetability [6,7,8,9]. In this respect, metal and metal oxide nanoparticles have been extensively researched for their photocatalytic, optoelectronic, bioengineering, magnetic, antimicrobial, wound-healing, and anti-inflammatory properties [10,11,12,13] arising from their unique physical and chemical characteristics, different from those of the bulk phase [14,15]. Among metal oxide nanoparticles, zinc oxide has extensive applications in various fields such as optics, piezoelectricity, bioimaging, and biosensing [16,17,18]. In particular, ZnO presents sufficient antimicrobial efficacy when the particle size is decreased to the nanometer range, as ZnO nanoparticles can interact with the bacterial core or surface as soon as they contact the cell and then exhibit distinct antibacterial mechanisms [19]. Therefore, ZnO nanoparticles are of great interest to biologists because of their excellent antibiotic properties and good biocompatibility. This distinct activity has opened new frontiers in the biological sciences. However, excessive ROS generated by ZnO nanoparticles may cause cell membrane disintegration, membrane protein damage, and genomic instability, which can initiate or enhance the development of many diseases [20,21]. Therefore, the development of novel multifunctional ZnO nanoparticles with antioxidant activity could minimize damage caused by excessive ROS.

In this study, we report a method for fabricating ZnO@GA nanoparticles by a simple surface modification process. The nanoparticles display strong antioxidant and antibiotic effects and were particularly effective against MRSA, suggesting that they can be a useful novel antibacterial agent for removing MRSA.

## 2. Results and Discussion

Antioxidant-functionalized ZnO@GA nanoparticles were synthesized via a simple coating of ZnO nanoparticles with gallic acid (GA). The ZnO nanoparticles were prepared by a sol-gel process. Then, the carboxyl group of GA was covalently bonded to surface Zn^2+^ ions, forming multifunctional ZnO@GA nanoparticles.

**Figure 1:**
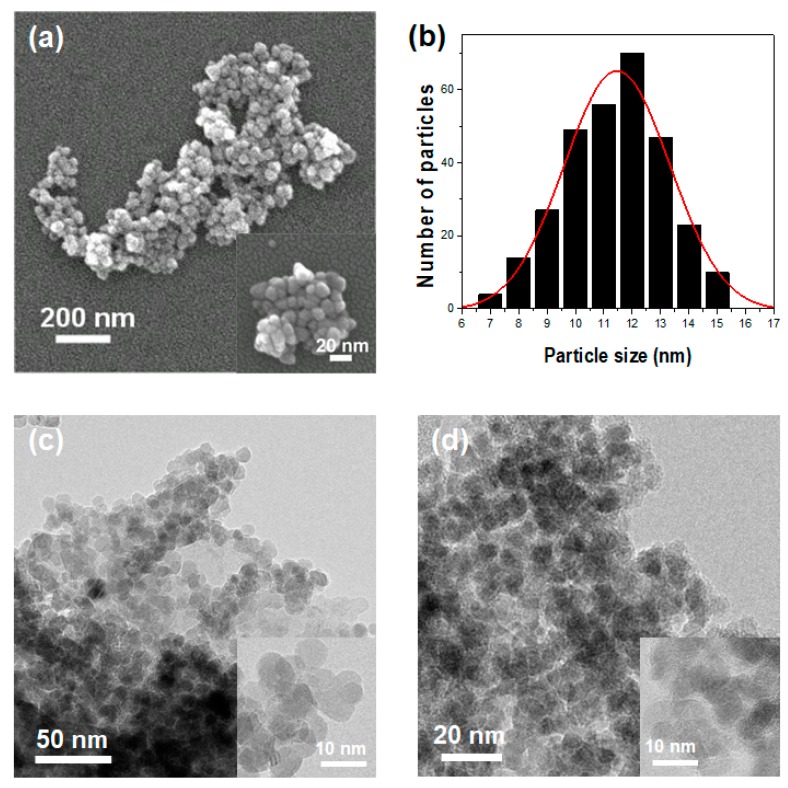
(**a**) Low-magnification FE-SEM image of the ZnO@gallic acid (GA) nanoparticles. A corresponding high-magnification FE-SEM image is shown in the inset; (**b**) Histogram of the ZnO@GA nanoparticle size distribution; TEM images of (**c**) ZnO nanoparticles and (**d**) ZnO@GA nanoparticles. Corresponding high-resolution TEM images are shown in the insets.

Pure ZnO and ZnO@GA nanoparticles were analyzed using various analytical tools. Field emission scanning electron microscopy (FE-SEM) confirmed that the ZnO@GA nanoparticles were nearly spherical in shape, with good size uniformity (Figure 1a). High-magnification scanning electron microscopy (SEM) images of the ZnO@GA nanoparticles showed a variety of spherical shapes (inset of Figure 1a), indicating that the nanoparticles aggregated spontaneously. Figure 1b indicates the size distribution of pure ZnO nanoparticles, which was estimated by sampling 300 particles in the Transmission electron microscopy (TEM) image. The average size of the ZnO nanoparticles was 11.5 ± 4.4 nm. TEM confirmed that ZnO and ZnO@GA nanoparticles both had globular shapes and assembled in aggregates on the TEM grids (Figure 1c,d). It is notable that there was almost no change in particle size or morphology after the surface modification. However, comparing the images in Figure 1c,d, the ZnO@GA nanoparticles appeared blurrier than pure ZnO nanoparticles owing to the conjugated antioxidant molecules.

**Figure 2:**
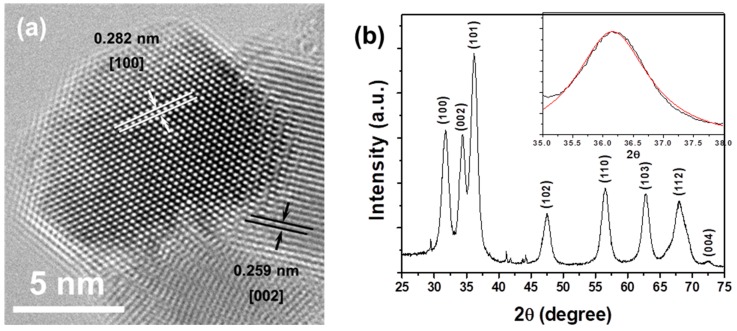
(**a**) High-resolution transmission electron micrograph of the ZnO@GA nanoparticles; (**b**) X-ray diffraction (XRD) pattern of ZnO@CA nanoparticles. a.u., arbitrary units.

High-resolution TEM (HR-TEM) and X-ray diffraction (XRD) analysis were used to obtain a more detailed crystal structure of the ZnO@GA nanoparticles. The selected area electron diffraction (SAED) pattern is presented in Figure 2a. The interlayer distances of the ZnO@GA nanoparticles were calculated to be 0.282 and 0.259 nm, which are comparable to the (100) and (002) planes of hexagonal ZnO, respectively [22]. This result indicates that each of the ZnO@GA nanoparticles has a single crystalline nature; thus, the ZnO@GA nanoparticles displayed high crystallization. Figure 2b shows the powder XRD data of the ZnO@GA nanoparticles. The strong Bragg reflection peaks (2θ = 31.7°, 34.4°, 36.1°, 47.6°, 56.5°, 62.8°, 67.9° and 72.1°), matched by their Miller indices ((100), (002), (101), (102), (110), (103), (112), and (004)), were obtained from a standard wurtzite ZnO structure (JCPDS Card No. 36-1451) [23]. Therefore, hexagonally structured ZnO was identified as a single crystalline phase in the ZnO@GA nanoparticles. The diffraction peak profile (2θ = 36.1°) was fairly well fitted by a convolution of Lorentzian functions (inset of Figure 2b). The mean crystalline size of the ZnO@GA nanoparticles was 5.8 nm, calculated based on Scherrer’s equation.

**Figure 3:**
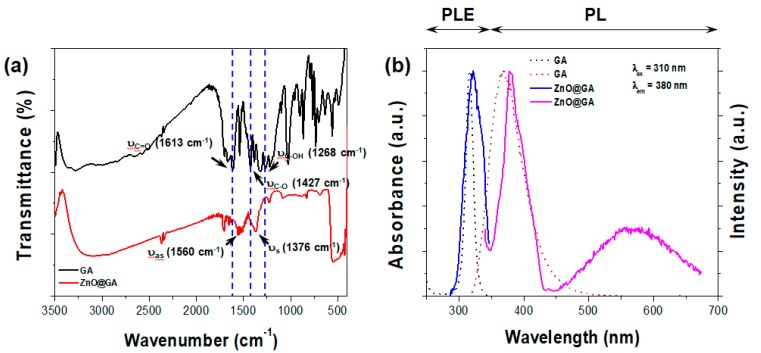
(**a**) Fourier-transform infrared spectroscopy spectra of pure GA molecules and ZnO@GA nanoparticles; (**b**) PL and PLE spectra of pure GA molecules and ZnO@GA nanoparticles in water. The excitation and detection wavelengths for both spectra were 310 and 380 nm. a.u., arbitrary units.

To confirm the binding between the carboxyl group of the GA molecules and Zn^2+^ cations on the surface of ZnO, Fourier-transform infrared (FT-IR) spectra of pure GA molecules and the ZnO@GA nanoparticles were compared (Figure 3a). The main peaks of the ZnO@GA nanoparticles and pure GA were very similar, resembling the characteristic peaks of GA. This indicates that GA molecules remain on the surface of ZnO nanoparticles even after washing with ethanol. In particular, both samples showed the presence of a carboxyl group (2700 to 3600 cm^−1^), hydroxyl phenolic groups (3284, 3382 cm^−1^), and an aromatic moiety (1541, 1618 cm^−1^), as shown in Figure 3a [24]. However, the pure GA molecules had absorption peaks at 1613, 1427, and 1268 cm^−1^, according to the stretching modes of the free carbonyl double bond (υ_C=O_), the C–O single bond (υ_C–O_), and the oxygen-hydrogen deformation (υ_C–OH_) (top panel of Figure 3a). This result indicates that pure GA molecules have protonated carboxyl groups (COOH), as expected. Conversely, the ZnO@GA nanoparticles displayed strong novel peaks at 1560 and 1376 cm^−1^. These new bands can be attributed to the asymmetric (υ_as_ = 1560 cm^−1^) and symmetric (υ_s_ = 1376 cm^−1^) stretching modes of the carboxyl group, as shown in Figure 3a (bottom panel). These results indicate that the carboxyl group bound to the surface of the ZnO nanoparticle.

Figure 3b shows the photoluminescence and photoluminescence excitation (PL and PLE) spectra of pure GA molecules and ZnO@GA nanoparticles. The peak at 315 nm is an absorption band typical of pure GA molecules, which may be attributed to the aromatic ring. Also, pure GA molecules had one strong emission peak, located at 368 nm (λ_ex_ = 310 nm). After surface modification, the PL and PLE spectra of the ZnO@GA nanoparticles exhibited characteristics very similar to those of pure GA molecules. However, the emission spectrum of the ZnO@GA nanoparticles also displayed emission at 450 to 650 nm. Broad, red-shifted emissions are typically observed with ZnO nanomaterials and are attributed to a recombination process through electronic states originating from oxygen vacancies or surface defects [25]. 

The antioxidant efficacies of pure GA molecules and ZnO@GA nanoparticles were evaluated using an ABTS radical scavenging method. The ZnO@GA nanoparticles contained an average of 2.89 GA molecules per particle and were suggested to have excellent diffusion and stability in water. The antioxidant activities of the ZnO@GA nanoparticles and pure GA are shown in Table 1. Pure GA molecules scavenged ABTS radicals proportionally to the concentration. The hydroxyl groups of GA are important for its free radical scavenging efficiency. In particular, the OH at the para-position to the carboxyl group appears to be essential for maintaining scavenging activity, as the scavenging activity is diminished by its methylation [26]. The ZnO@GA nanoparticles also robustly scavenged ABTS radicals. The decrease in the antioxidant activity of the ZnO@GA nanoparticles compared to that of pure GA molecules may be attributable to steric repulsion between the nanoparticles and ABTS radicals. 

**Figure 4:**
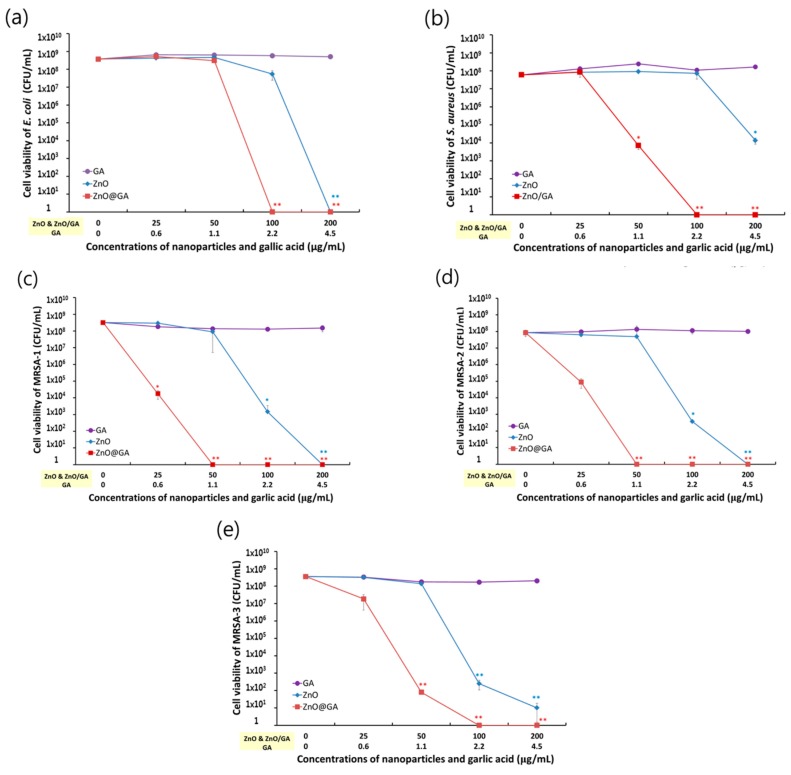
Antibacterial effects of ZnO@GA nanoparticles. (**a**) *E. coli*; (**b**) *S. aureus*; (**c**) MRSA-1; (**d**) MRSA-2; and (**e**) MRSA-3. Data are shown as the mean ± S.D (*n* = 6). Analysis of statistical significance (* *p* < 0.05, ** *p* < 0.005 versus control) was performed using Student’s *t*-test.

**Figure 5:**
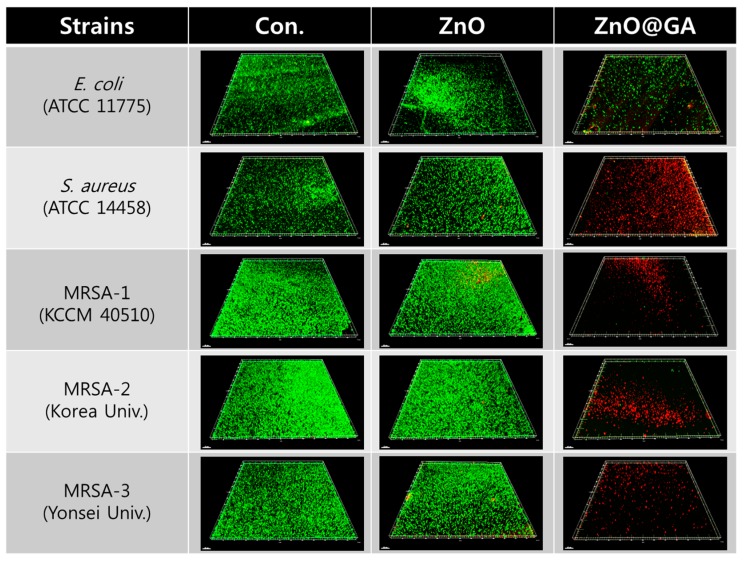
Qualitative assay of antibacterial activity using live and dead cell staining of Gram-negative and Gram-positive bacteria. Fluorescent images show live cells stained by SYTO-9 (green) and dead cells stained by PI (red) after 24 h of incubation. Scale bars represent 50 μm.

To confirm the antibacterial effects of GA, ZnO, and ZnO@GA nanoparticles, a static culture method was used after mixing the bacteria and nanoparticles, as previously described [27]. The antibacterial effect of each sample was tested in five bacterial strains (a Gram-negative strain, i.e., *E. coli*, and four Gram-positive strains, i.e., one *S. aureus* and three MRSA). The antibacterial activities of each sample were evaluated by counting the colony-forming units (CFUs) of each strain as a measure of the total number of viable bacteria (Figure 4). The ZnO@GA nanoparticles showed strong antibacterial activity, two to four fold higher than that of ZnO nanoparticles, and displayed higher antibacterial activity against *S. aureus* and MRSA than against *E. coli*. The ZnO@GA nanoparticles at 50 and 100 μg/mL completely inhibited MRSA-1 and MRSA-2 strains, and they more effectively killed the MRSA strains than the *S. aureus* strain at 50 μg/mL, suggesting that ZnO@GA nanoparticles are specifically effective against MRSA strains. The selective inhibition effects were also confirmed using confocal fluorescence microscopy, as shown in Figure 5. The confocal fluorescence microscopy showed that the ZnO@GA nanoparticles have strong killing effects against Gram-positive bacteria, with complete killing of the cells in the *S. aureus* and MRSA samples. The viability of Gram-negative *E. coli* decreased as well, although many cells remained viable. This result also confirmed that the ZnO@GA nanoparticles have selective inhibitory activity against Gram-positive bacteria and particularly against MRSA. 

As shown in Figure 4, although the bactericidal effects were relatively weak compared to those of the ZnO@GA nanoparticles, the ZnO nanoparticles also showed inhibitory effects against the five strains at 100 and 200 μg/mL. GA is known to have antibacterial activity, with a minimum inhibitory concentration of 8 mg/mL for *S. aureus* [28]. However, in this study, GA was used at 0.6 to 4.5 μg/mL. At these concentrations, GA alone did not display antibacterial activity against the bacterial strains (Figure 4). 

Although GA treatment alone was not effective, the ZnO nanoparticles conjugated with low concentrations of GA, relatively lower concentrations than those in other published reports [28,29,30,31], had enhanced antibacterial properties compared to the ZnO nanoparticles, dramatically reducing the cell viability of Gram-positive bacteria, particularly MRSA. The strong antibacterial activity and selectivity may be attributed to the high affinity of GA for the bacterial cell membrane and the increased lipophilicity upon the addition of GA [31].

Overall, the results in this study suggest that ZnO nanoparticles functionalized with GA have antioxidant activity, as well as selective antibacterial activity, against MRSA. However, future studies will be required to evaluate their efficiency and bio-safety in vitro and in vivo.

## 3. Materials and Methods

### 3.1. Preparation of ZnO@GA Nanoparticles

ZnO nanoparticles were prepared by a process similar to that in a previous report [23]. A stock solution of Zn(CH_3_COO)_2_·2H_2_O (0.1 M) was dissolved in 50 mL of methanol under vigorous stirring. Then 25 mL of NaOH (0.2 M) in methanol was added to this mixture, and the pH was maintained at 8. This mixture was transferred to a Teflon-lined sealed stainless steel autoclave and maintained at 80 °C for 10 h. The resultant white solid products were washed with methanol several times, filtered, and then dried in a vacuum oven at 60 °C for 6 h.

A wet chemical process with GA was used to provide ZnO nanoparticles with antioxidant functionality, as follows. First, 20 mg of ZnO nanoparticles was diffused in EtOH (1 mL). This solution was added to a 2.08 × 10^−5^ M solution of GA/EtOH, and vigorous stirring was applied for 24 h. The resulting product was washed several times in EtOH and dried at 60 °C.

### 3.2. Physical Characterization of ZnO@GA Nanoparticles

FE-SEM was performed on a SU-70 Analytical UltraHighResolution SEM (Hitachi, Tokyo, Japan). TEM SAED and high-resolution (HR) TEM were performed with a JEM-3100F TEM (JEOL, Tokyo, Japan) operating at 200 kV. TEM samples were prepared by placing a drop of sample suspension onto a standard carbon-coated copper grid. This grid was dried before the micrographs were recorded. The phase structures of the sample were identified by XRD using a X’Pert Pro MPD (PANalytical, Almelo, The Netherlands) with CuKα radiation (wavelength of the radiation, *k* = 1.54 Å). *IR* spectra were obtained using a FT-IR spectrometer (Perkin-Elmer PE 100, Waltham, MA, USA). For IR measurements, the samples were crushed on mortar and then prepared as pressed wafers (1% sample in KBr). The PL and PLE of the samples were measured on a F-4500 spectrofluorimeter (Hitachi, Tokyo, Japan), using a Xe arc lamp (150W, Abet Technologies, Milford, CT, USA) as the excitation source.

### 3.3. Evaluation of ZnO@GA Nanoparticle Antioxidant Activity

The radical cation decolorization assay determines the capacity of substances to scavenge ABTS [31]. The ABTS radical cation (ABTS ^+^) was prepared by mixing 2.45 mM potassium persulfate and 7 mM ABTS stock solution (1/1) and was placed in a dark place until the absorption peak was stabilized. After 20 h, this solution was diluted with MeOH, and the absorbance was maintained at 1 (λ_max_ = 734 nm). For photo-detection, 0.9 mL of ABTS·^+^ solution was combined with 0.1 mL of different concentrations of GA and ZnO@GA nanoparticles, and mixed for 45 s. Measurements were taken at 734 nm after 15 min. The antioxidative activities of GA and ZnO@GA nanoparticles were estimated by detecting the decrease in absorbance at different concentrations using the following equation:

*E* = (*A*_c_ − *A*_s_/*A*_c_) × 100 (1)

where *A*_s_ and *A*_c_ are the respective absorbance of the samples and ABTS·^+^, expressed in μmol.

### 3.4. Evaluation of ZnO@GA Nanoparticle Antibacterial Activity

To evaluate the antibacterial effects of the ZnO@GA nanoparticles against a Gram-negative and four Gram-positive bacterial strains, we used previously described methods and bacterial strains [27]. Briefly, five bacterial strains were used: a Gram-negative bacteria, *E. coli* (ATCC 11775), and four Gram-positive bacterial strains, which were clinically isolated strains, namely, *S. aureus* (ATCC 14458), MRSA-1 (KCCM 40510), and MRSA-2 and MRSA-3, [32,33]. 

For the quantitative antibacterial test, *E. coli* and *S. aureus* were suspended at 10^6^ to 10^7^ CFU/mL in nutrient broth, and the MRSA strains were suspended in brain heart infusion (BHI) broth. After the 10-fold dilution of each bacterial suspension, the bacterial cells (approximately 10^5^ to 10^6^ CFU/mL) were inoculated in 24-well plates and incubated with various concentrations (0, 25, 50, 100, and 200 μg/mL) of GA, ZnO, and ZnO@GA samples at 35 °C. After 24 h of incubation, the bacterial cells in 24 well plates were diluted by a serial 10-fold dilution method and inoculated onto a plate count agar (PCA) plate for *E. coli* and *S. aureus* and a BHI agar plate for the MRSA strains. The PCA and BHI plates were incubated for a further 24 h, and the CFUs in each plate were counted. For the antibacterial activity of each sample, each bacterial cell viability was expressed as CFU/mL versus the concentrations of each sample, and an inhibition of >3 log in the cell viability of each strain was defined as a positive antibacterial effect.

For fluorescence microscopy to confirm the antibacterial effects of the samples, each bacterial cell type at 10^6^ to 10^7^ CFU/mL was plated onto a cover glass coated with poly-L-lysine and incubated for 1 h. Next, the cover glass was washed with 0.9% saline solution three times to remove the detached cells from the glass, before incubation with samples for 24 h. Finally, live and dead bacterial cells were stained with a LIVE/DEAD BacLight Bacterial Viability Kit (Molecular Probes, Eugene, OR, USA) according to the manufacturer’s instructions. Each bacterial cell was analyzed using a confocal fluorescence microscope (FV-1200, Olympus, Tokyo, Japan) with a 20× objective lens, excitation filters at 485 nm for both SYTO 9 and propidium iodide (PI), and emission filters at 530 and 630 nm for SYTO 9 and PI, respectively. Each fluorescence image was analyzed using imaging software (Imaris, Bitplane, Concord, MA, USA).

### 3.5. Statistical Analysis

The experiments were repeated three times (*n* = 6), and the quantitative data are shown as the mean ± standard deviation (S.D.). The statistical significance (*p* < 0.05) was analyzed by Student’s *t*-test. 

## 4. Conclusions

In this study, we successfully fabricated multifunctional ZnO nanoparticles conjugated with GA via a simple surface modification process. These multifunctional ZnO@GA nanoparticles show high antioxidant and antibacterial activity, and the functionality and potentiality on antioxidant and antibacterial activity suggest that they can be useful as a novel antibacterial agent for MRSA.

## Figures and Tables

**Table 1:** ABTS radical scavenging activity.

Sample	Concentration (μM)	% Inhibition
ZnO@GA	20	33.29 ± 0.12
ZnO@GA	40	57.17 ± 0.96 ^a^
ZnO@GA	100	69.71 ± 5.26 ^a^
Gallic acid	20	43.38 ± 0.48 ^b^
Gallic acid	40	72.76 ± 0.12 ^b^
Gallic acid	100	93.25 ± 0.43 ^b^

Data are expressed as the mean ± standard deviation (SD) of independent experiments (*n* = 3). Statistical significance (*p* < 0.05) was analyzed using a one-way analysis of variance with a post-hoc Tukey’s test. ^a^ Activity at the lowest concentration of the same sample. ^b^ Two different samples at the same concentration.
